# Seasonal Diet Composition of Goitered Gazelle (*Gazella subgutturosa*) in an Arid and Semi-Arid Region of Western China

**DOI:** 10.3390/ani14050663

**Published:** 2024-02-20

**Authors:** Nan Zhang, Zhirong Zhang, Chao Liu, Zeqin Xiong, Yaoyun Wei, Dehuai Meng, Meiling Zhan, Zongzhi Li, Yao Zhao, Liwei Teng, Zhensheng Liu

**Affiliations:** 1 College of Wildlife and Protected Areas, Northeast Forestry University, Harbin 150040, China; zhangnan4292022@163.com (N.Z.);; 2 Luoshan National Nature Reserve Management Bureau of Ningxia Hui Autonomous Region, Wuzhong 751999, China; 3 Key Laboratory of Conservation Biology, National Forestry and Grassland Administration, Harbin 150040, China

**Keywords:** diet, goitered gazelle, microhistology, seasonal diet

## Abstract

**Simple Summary:**

Understanding the resource requirements, including the dietary needs, of threatened species is crucial for formulating effective conservation strategies, especially in ecologically sensitive arid and semi-arid regions. In recent years, there has been a significant decline in the population of goitered gazelles, a representative species in arid and semi-arid regions. Therefore, the aim of our study was to evaluate the components and seasonal variation in diet of the goitered gazelle. The primary foods of the goitered gazelles included Chenopodiaceae, Rosaceae, Gramineae, Asteraceae, and Fabaceae. Furthermore, there was seasonal variation in the food composition and dietary preferences of the species. Based on these findings, we propose conservation recommendations for goitered gazelles and other herbivores living in arid and semi-arid regions.

**Abstract:**

Global climate change, habitat fragmentation, and human interference have resulted in a significant, ongoing decline in the population of goitered gazelles. Effective conservation strategies require an understanding of resource requirements of threatened species, such as dietary needs. Therefore, we aimed to elucidate the food composition and seasonal dietary changes of goitered gazelles through microhistological analyses of fresh feces. Fabaceae (11.5%), Gramineae (9.4%), Chenopodiaceae (20.2%), Asteraceae (10.1%), and Rosaceae (19.5%) formed the primary dietary components of goitered gazelle. Additionally, *Krascheninnikovia arborescens* (13.4%) and *Prunus sibirica* (16.3%) were identified as the key forage plants. Forbs (50.4%) were the predominant plants for grazing throughout the year, particularly in the spring (72.9%). The proportion of trees in the diet was highest in the autumn (36.7%) and comparatively lower in other seasons. Furthermore, the proportions of shrubs (22.0%) and graminoids (14.8%) both reached their peaks in the winter. Our findings indicate that goitered gazelles strategically forage seasonally to cope with resource bottlenecks, enhancing their adaptability to arid and semi-arid habitats. Our study provides essential ecological information for the conservation of goitered gazelles and emphasizes the importance of dietary studies of species of ecological significance in environmentally sensitive areas.

## 1. Introduction

Understanding the ecology of herbivores is crucial for developing strategies that promote their sustainability [1]. As a fundamental aspect of ecological research, diet contributes to the substantial body size variation observed in herbivore populations [2] and provides valuable basic information for estimating environmental capacity [3], calculating energy metabolism [4], assessing habitat suitability [5], analyzing intra- and interspecific relationships [6], and formulating protection strategies [7]. Additionally, seasonal dietary changes play a significant role in the avoidance of seasonal resource bottlenecks by herbivores [8,9,10]. There is evidence that flexibility in seasonal diet contributes to herbivore survival under drought and facilitates population growth [9,11]. Therefore, seasonal dietary strategies are particularly crucial for herbivores residing in arid and semi-arid regions.

Goitered gazelle, belonging to the family Bovidae, play a crucial ecological role in arid and semi-arid regions [12]. The species has been listed as a national key protected animal of class II in China [13]. It is solitary or occurs in small groups of 2–10 individuals [14]. Historically, goitered gazelle were widely distributed from the Arabian Peninsula to southern Mongolia [15,16]. Since the beginning of the 20th century, the range has been diminishing [12,17,18] and the species is now mainly found in Mongolia, northwestern China, Russia, Iran, and Pakistan, and among other locations [19]. In 2001, the population of goitered gazelle was estimated to be between 120,000 and 140,000 individuals [20]. But anthropogenic disturbances, habitat loss, and the impacts of climate change have resulted in a substantial decline in the population [13,19,20,21,22]. In 2016, the population was estimated to be within the range of 42,000 to 49,000 individuals [23]. Furthermore, the species has been officially declared extinct in Georgia, Armenia, Kuwait, and Qatar [24].

Due to the previous relative abundance of goitered gazelles [15,16,20], they were assigned a relatively lower priority in research. Consequently, studies on the dietary habits of goitered gazelles are still limited. In Bahrain, the main food sources for goitered gazelle have a high water content or are rich in protein [25]. Another study reported that goitered gazelle in the Ibex Reserve of Saudi Arabia predominantly consume woody plants [26]. In the study conducted in the Karameili Mountains of Xinjiang, China, two forbs were determined as the principal dietary components, exhibiting discernible seasonal variations in dietary patterns [27,28]. Our study contributes to a deeper understanding of goitered gazelles’ utilization and adaptability to food resources across different seasons. This knowledge is crucial for the more effective conservation and management of this species.

Here, we aimed to investigate the annual dietary composition and seasonal dietary strategies of goitered gazelle. We hypothesized that forbs were the main dietary component of the species, with food preferences and the degree of selection differing among seasons. The results of our study provide key information for the effective conservation and management of endangered herbivores inhabiting arid and semi-arid ecosystems.

## 2. Materials and Methods

### 2.1. Study Area

**Figure 1:**
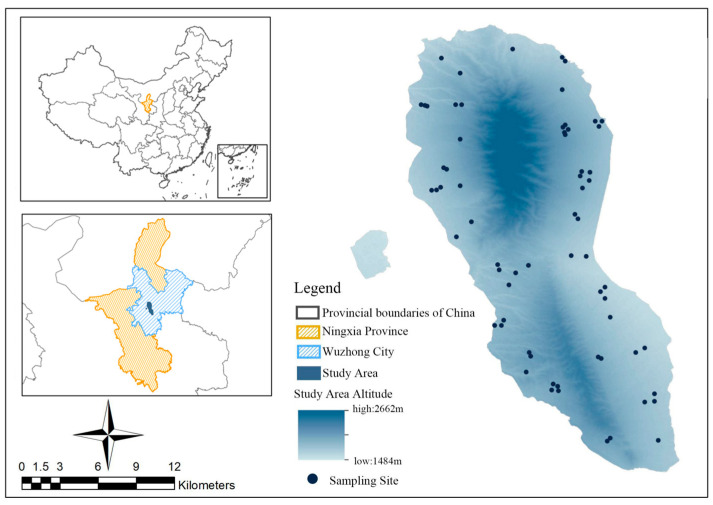
Location of the study area and sampling sites in our study. The left-hand side inserts show the location of the Luoshan National Nature Reserve in Wuzhong City, Ningxia Hui Autonomous Region, China. The main map shows the study area with the sampling sites.

Our study was conducted within the Luoshan National Nature Reserve (37°11′–37°25′ N, 106°04′–106°24′ E), in the central part of the Ningxia Hui Autonomous Region, China (Figure 1) [29]. Covering an expansive area of 337.1 km^2^, the reserve encompasses two mountain ranges aligned in a north–south direction. The topography of the reserve is characterized by undulating terrain, with elevations ranging from 1560.0 to 2624.5 m above sea level (a.s.l.) [30]. There are 38 gullies of various sizes within the area, with seasonal water flow. The dry season is relatively arid, while the rainy season sees swift water flow in the rivers [31]. This unique ecological setting provides a diverse and complex landscape for the study of goitered gazelle and their interactions within this habitat.

### 2.2. Climate

The study area typifies a temperate continental climate, characterized by four distinct seasons [29]. The annual average temperature is 8.8 °C, with an extreme minimum temperature recorded at −27.1 °C. The coldest month is January, featuring an average monthly temperature below −9 °C, whereas July is the warmest month, with an average monthly temperature rising to 24 °C. Notably, the area exhibits a diurnal temperature variation of 10.2 °C. The annual average evaporation is 2467.3 mm, which is 9.4 times the average annual precipitation of 261.8 mm [32].

### 2.3. Flora and Fauna

There are 506 species of vascular plants, taxonomically classified into 73 families and 244 genera, in the study area. Predominant families include Asteraceae, Gramineae, Leguminosae, and Rosaceae, while prevailing genera are Artemisia and Astragalus. The distribution of vegetation can be divided into four distinct zones [33]: (1) The Qinghai spruce forest zone (>2500 m a.s.l.) is characterized by Qinghai spruce (*Picea crassifolia*), Ladybell root (*Adenophora stricta*), and Halberd (*Euphorbia pekinensis*). (2) The Pine-Aspen-forest zone (2150–2500 m a.s.l) predominantly includes Chinese pine (*Pinus tabuliformis*), Aspen (*Populus davidiana*), Birch (*Betula platyphylla*), Hazelnut (*Ostryopsis davidiana*), and other associated plants. (3) The shallow mountain shrub zone (<2150 m a.s.l) features common plant species, such as Berberis, Caragana, Cotoneaster, Ostryopsis, and Spiraea. (4) Foothill steppe zone (<2100 m a.s.l.) primarily includes Cat head thorn (*Oxytropis aciphylla*), Tarragon (*Artemisia frigida*), and Pigweed (*Kali collinum*), among other plants [34].

The protected area is characterized by rich wildlife resources, with 218 known vertebrate species distributed across 67 families and 141 genera. Prominent among these species are the Golden eagle (*Aquila chrysaetos*), Chinese mountain cat (*Felis bieti*), Eurasian lynx (*Lynx lynx*), Stone marten (*Martes foina*), Pallas’s cat (*Otocolobus manul*), Common kestrel (*Falco tinnunculus*), and Goitered gazelle (*Gazella subgutturosa*) [35].

### 2.4. Plant Diversity

Since goitered gazelle in the study area are distributed only in the foothill steppe zone and shallow mountain shrub zone at an elevation of less than 2150 m, it is necessary to collect plant diversity data specifically for the shrub and grass communities. According to the literature [36], the shrub and grass communities in the Luoshan region include 112 plant species belonging to 86 genera and 41 families. These include 22 woody species, accounting for 19.6% of the total plant species, and 90 herbaceous species, accounting for 80.4% of the total. Dominant plant families include Asteraceae (15.2%), Fabaceae (11.6%), Rosaceae (11.6%), and Gramineae (10.7%). These four families collectively include 55 plant species, representing 49.1% of the total. Dominant genera include Artemisia (6.3%), Astragalus (3.6%), and Potentilla (3.6%). There are 70 monotypic genera, constituting 62.5% of all genera. Dominant species include North China kochia (*Krascheninnikovia arborescens*), *Kali collinum*, Sedge (*Cyperus rotundus*), Myricaria (*Caragana korshinskii*), Thorny spiraea (*Convolvulus tragacanthoides*), and the Gammon golden pheasant (*Caragana opulens*).

### 2.5. Sample Collection

**Figure 2:**
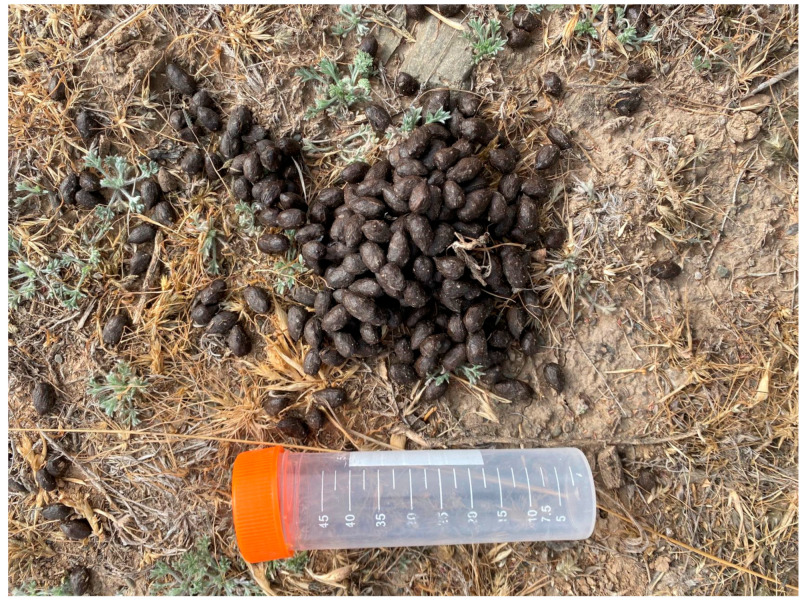
The feces of goitered gazelle.

Samples were collected from October 2019 to September 2020, divided into spring (March–May), summer (June–August), autumn (September–November), and winter (December–February) [29]. Fresh fecal samples, distinguished by a smooth surface and dark hue [37], were collected opportunistically from known and potential goitered gazelle distribution areas in the reserve. Owing to the absence of other ungulates in the reserve, the pellet-like feces of goitered gazelle are easily distinguishable (Figure 2). All pellets from a single defecation event were taken as one sample. The position of each sample collection point was recorded (Figure 1). These samples were then frozen at −20 °C for subsequent experiments. Overall, 152 fecal samples were collected: 38 in the spring, 32 in the summer, 40 in the autumn, and 42 in the winter.

Simultaneously with fecal sample collection, a 10 m × 10 m quadrat was centered around the fecal deposition site to collect plant species that goitered gazelle might have consumed. Two samples were collected for each plant species (one used as a plant specimen to prepare reference slides, and the other for species identification and as a reserve sample, preserved by drying). In total, 58 plant species were collected.

### 2.6. Microhistology

Fecal microhistological analysis is a commonly used method for analyzing the feces of herbivores to determine their dietary composition using histological techniques [38,39,40,41,42,43,44]. The fundamental principle involves identifying the plant types and quantities consumed by animals based on the imprinted structure of undigested phytokeratin fragments present in the feces [45]. Various methods are employed for microscopic examination, including direct counting, line interception, area, point sampling, and frequency conversion. Among these, the frequency conversion method stands out as the simplest and most expedient [46]. Fecal microhistological analysis benefits from easy sample acquisition and preservation and minimal disturbance to wild animals. Compared to the recent emerging DNA barcoding technology, a disadvantage is the substantial workload, and the results can be influenced by factors such as the subjective cognitive differences in plant cuticle cell recognition methods and personnel. The advantage is the lower cost and simplicity of procedures, allowing avoidance of the impact of some uncontrollable factors on the accuracy of analysis (e.g., the degradation of DNA extracted from fecal samples) [47].

The basic experimental steps involve the preparation of reference slides derived from diverse plant components, including leaves, stems, flowers, and fruits. These reference slides are then systematically compared with slides generated from fecal samples obtained from the target species [45,48]. The microscopic identification of forage plant fragments was conducted using the taxonomic criteria delineated by Satkopan [31] and Johnson et al. [49].

Plant samples were dehydrated in a Baihui 101-3B oven at 120 °C for 48 h, ground at 1 mm in a Willey mill, and sieved through 40-mesh (0.425 mm) and 100-mesh (0.150 mm). Approximately 1 g of the supernatant from the 100-mesh sieve was placed in a Petri dish and immersed in a 10% sodium hypochlorite solution for 4–8 h. Temporary slides were prepared and a microscope was used to observe whether the cell morphology was clear. If the morphology was not clear, the immersion time was extended. After achieving a clear cell morphology, saffron red staining for ~30 min, followed by stain removal, was used for microscopic mount preparation [1,45,50,51]. Each plant specimen was used to prepare five slides. These slides, viewed under a Leica DM 1000 orthostatic microscope at 100× magnification, captured fields with distinct plant cells, forming a reference library. The preparation method for fecal microscopic slides was similar to that for plant slides, except immersion in a 10% sodium hypochlorite solution was required for only 2–4 h.

Ten slides per fecal sample were prepared, with images of 100 non-duplicate visual fields obtained at 100× magnification. A visual field was deemed valid if it contained at least one identifiable plant fragment [1,52]. Identification up to the species level was attempted by comparing each sample with the reference plant library whenever possible. The microscopic readings were expressed as relative frequencies. The identified plant species were categorized into four primary functional groups: forbs, graminoids, trees, and shrubs.

### 2.7. Data Analyses

The data were processed using the frequency conversion method [53]. Statistical calculations were performed to determine the occurrence frequency (*F*) of cuticular debris for each plant. The average density of identifiable epidermal cuticle fragments for each plant in every field of view was calculated. The resulting density (*D*) was then converted to a relative density (*RD*), which represents the dry weight composition ratio of different plants in the diet.

*F* = 100(1 − *e*^−*D*^)

*D* = −In(1 − *F*/100)

*RD* = *D_i/_*∑*D_i_*

Food diversity of goitered gazelle was described using the Shannon–Wiener index (*H*) [54]. The proportion of plant fragments of species *i* out of all plant fragments was designated as *P_i_*, while the number of plant species in the fecal sample was identified as *S*.

$$H=-\sum_{i=1}^{S}P_{i}\log P_{i}$$

The width of trophic ecological niche (*B*) of goitered gazelle was calculated based on the following equation [55]:

$$B=1/\sum P_{i}^{2}$$

## 3. Results

### 3.1. Dietary Composition of Goitered Gazelles

**Figure 3:**
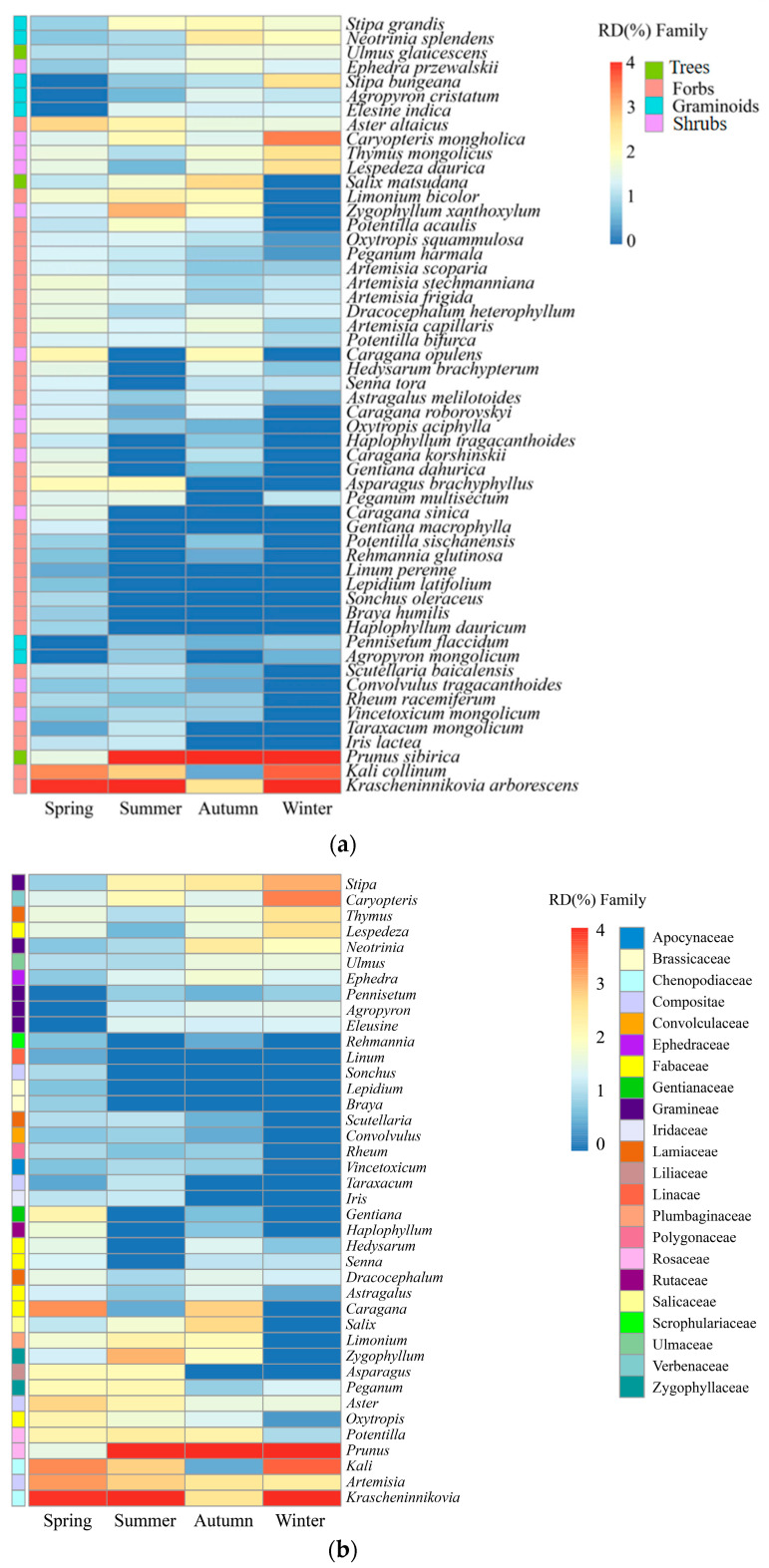
Dietary composition of goitered gazelles across four seasons, showcasing the proportion of plant species and functional groups (**a**), and the proportion of genera and families (**b**).

Throughout the year, 54 species of plants were consumed by goitered gazelles belonging to 22 families and 40 genera. Fabaceae, Gramineae, Chenopodiaceae, Asteraceae, and Rosaceae formed the primary dietary components, which collectively accounted for 66.2–78.0% of their annual food intake. Forbs were the predominant component, making up 50.4% of the diet, highlighted by *Krascheninnikovia arborescens* (13.4%) and *Kali collinum* (6.8%). Trees held secondary importance at 20.3%, with *Prunus sibirica* contributing an average of 16.3%. Apart from *Krascheninnikovia* (13.4%) and *Prunus* (16.3%), *Kali* (6.8%), *Stipa* (4.2%), and *Caryopteris* (4.2%) were also components of the diet (Figure 3a,b; Table S1).

**Figure 4:**
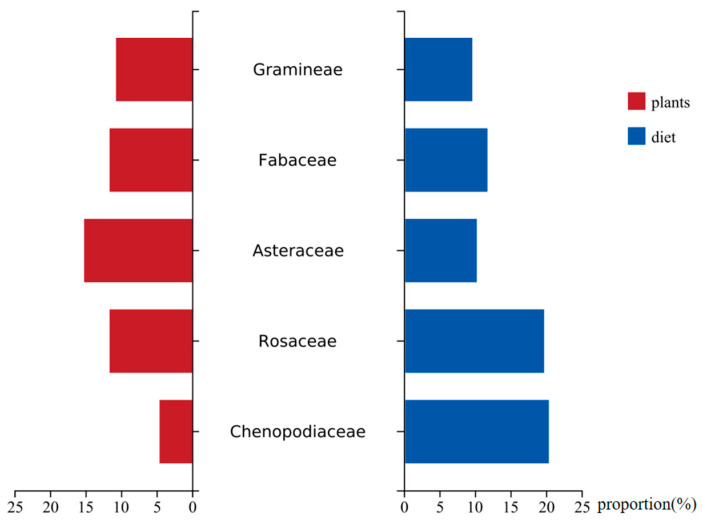
Proportion of goitered gazelles’ primary food in the diet and proportion availability in the study site.

The proportions of Fabaceae (11.5%) and Gramineae (9.4%) in the diet of goitered gazelles were similar to the available proportions of these two plant families in the study area. The availability of Asteraceae (15.2%) exceeded the proportion in goitered gazelle diet (10.1%). The proportions of Rosaceae (19.5%) and Chenopodiaceae (20.2%) in the diet were higher than their respective plant availability, especially for Chenopodiaceae (4.6%) (Figure 4). Additionally, the primary foods of goitered gazelles, *K*. *arborescens* and *K*. *collinum*, are dominant species, while *P*. *sibirica* is not.

### 3.2. Seasonal Variation in the Diet of Goitered Gazelles

In the spring, goitered gazelles consumed plants from 22 families and 49 species; in this season, the food diversity index *H* (1.54) and food niche width *B* (21.67) were highest. During the autumn, goitered gazelles consumed 42 plant species from 18 families, surpassing the summer counts of 39 plant species from 17 families. The food niche width *B* values were lower in the autumn (9.82) than in the summer (13.94), while the food diversity index *H* was similar between the two seasons. In the winter, 28 plant species from 10 families were consumed, representing the lowest food diversity index *H* (1.31) and food niche width *B* (6.76) among all seasons (Table 1).

**Figure 5:**
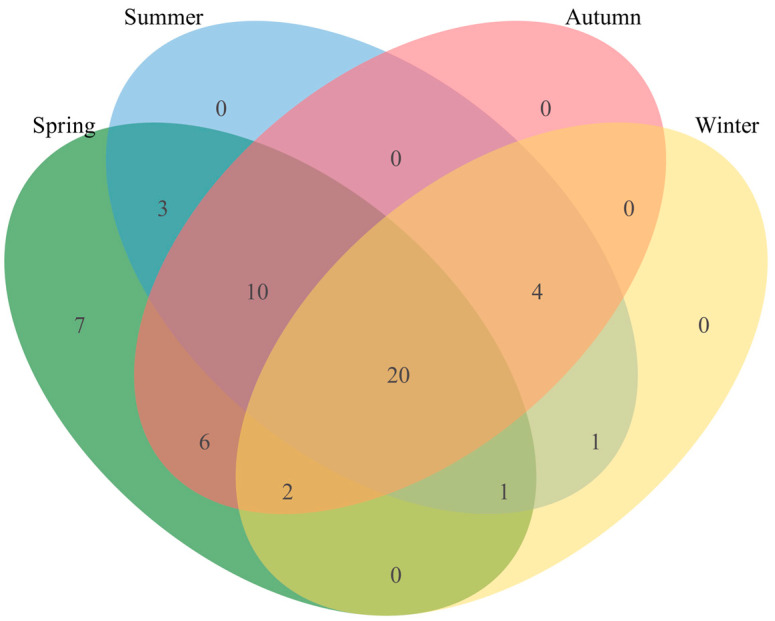
Seasonal food overlap of goitered gazelles. The green part represents the food composition of spring. The blue part represents the summer. The pink part represents the autumn. The yellow part represents the winter. Numerical values represent the quantity of plant species shared or unique in the dietary composition across various seasons.

Excluding the 20 plant species consistently consumed throughout the year, which included five of the seven staple foods, the relative proportions of major plant species in the goitered gazelle diet exhibited seasonal variation. *K. arborescens*, *K*. *collinum*, *Aster altaicus*, and *Caragana opulens* formed the primary dietary components in the spring, in addition to seven unique plants that were present but were not major food sources (Figure 5). In the summer, *P. sibirica*, *K. arborescens*, *Zygophyllum xanthoxylum*, and *K. collinum* were the primary food sources. In the autumn, *P. sibirica*, *Salix matsudana*, *K. arborescens*, and *Neotrinia splendens* formed the main dietary components. In the winter, the primary food sources were *P. sibirica*, *K. arborescens*, and *K. collinum*, which accounted for 47.8% of the total food composition (Table 2).

**Figure 6:**
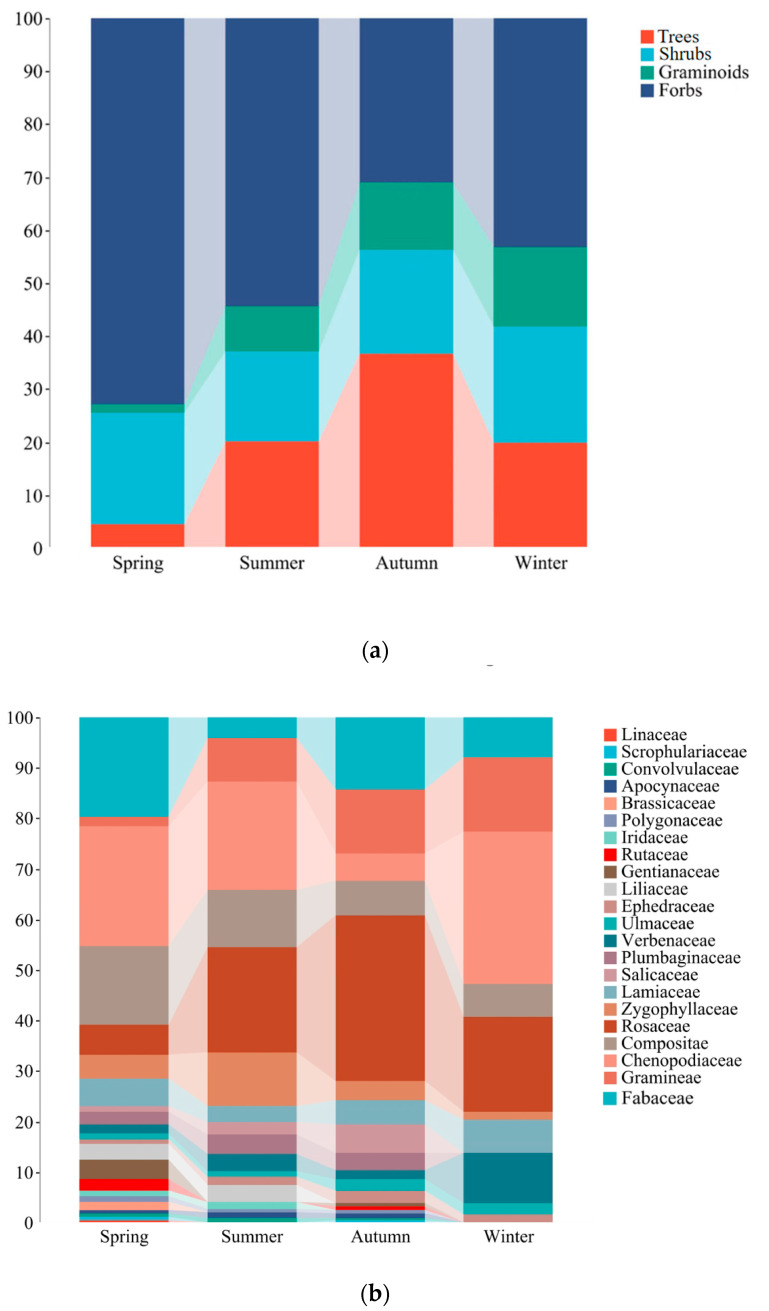
Seasonal dietary contribution by plant categories (**a**) and families of plants (**b**) to the diet of goitered gazelle.

Forbs consistently played a crucial role throughout the year, peaking at 72.9% of the diet in the spring. Trees showed the highest contribution in the autumn (36.7%), with lower contributions in other seasons. Shrubs exhibited minimal variation across seasons but displayed an increasing trend from summer (17.1%) to winter (22.0%). Similarly, graminoids demonstrated an upward trend from spring to winter, increasing from 1.6% to 14.8% in the diet of goitered gazelles (Figure 6a).

Fabaceae plants exhibited a significantly higher contribution to the diet in the spring and autumn (19.8% and 14.3%) than in the summer and winter (4.2% and 7.9%). Chenopodiaceae plants demonstrated the lowest dietary contribution in the autumn (5.3%) and higher levels in other seasons, particularly in the winter (30.1%). Compositae plants displayed a decreasing trend from spring to winter, ranging from 15.7% to 6.5% in the diet of goitered gazelles, with similar estimates in the autumn (6.9%) and winter. Rosaceae plants played a pivotal role in autumn, reaching 32.7%. Additionally, plants in Linaceae (0.4%) and Brassicaceae (1.6%) were exclusively detected in the spring diet (Figure 6b).

## 4. Discussion

### 4.1. Dietary Composition of Goitered Gazelle

The diet of goitered gazelle from an arid and semi-arid area in China was analyzed. The results showed that goitered gazelle primarily consumed plants in the families Fabaceae, Gramineae, Chenopodiaceae, Asteraceae, and Rosaceae. The preferences for Fabaceae, Gramineae, Asteraceae, and Rosaceae can be attributed to their dominant status in the protected area, providing a relatively large biomass to fulfill the dietary needs of the species. The preference for Chenopodiaceae can be explained by its relatively high crude protein and fat contents, satisfying the dietary requirements of goitered gazelles [56,57]. This aligns with the dietary habits of goitered gazelles in Xinjiang, China [27,28]. Additionally, they consumed abundant *Krascheninnikovia* and *Kali*, possibly due to their high water content. A study on water intake in goitered gazelles suggests that they adapt to arid and semi-arid environments by selecting food with high water contents [58]. Similar use of berries as a water source was seen for ungulates in arid environments in Canada [59].

In our study, *K*. *arborescens* and *P*. *sibirica* were the main food sources for goitered gazelles, with the former attributed to the high diversity of *K. arborescens*. Despite the lower plant abundance of *P. sibirica*, goitered gazelles consumed it in large quantities, likely due to the high water content in its leaves. Notably, *P. sibirica* was not recorded in the diet of goitered gazelles previously [25,26,27,28,60], which could be attributed to variation in plant diversity between research areas and the prevalence of dwarf apricot trees in Luoshan, affecting their availability. In eastern Xinjiang’s Mulei County, goitered gazelles rely primarily on *Ephedra* as a food source [60]. However, in our study, *Ephedra* constituted only a small fraction of goitered gazelle diet. Furthermore, *Ephedra* was not detected in the feces of goitered gazelles in three seasons at Karamali Mountain in Xinjiang [61]. *Ephedra* is distributed in all three regions, with similar accessibility [36,62,63]. This discrepancy may be due to significant temperature differences between the study areas. Mulei County, located in the eastern part of the Junggar Basin, serves as a passage for cold fronts and is the coldest area in the winter among regions at the same latitude in China [64]. To cope with the cold climate, goitered gazelles may forage *Ephedra*, which has beneficial effects on cold fronts in mammalian Chinese medicine [65]. However, goitered gazelles in Luoshan do not face such extreme cold conditions. Coupled with the relatively low palatability of *Ephedra*, they consume minimal amounts of this plant.

### 4.2. Seasonal Variation in the Diet of Goitered Gazelles

Our study demonstrated that there is pronounced seasonal variation in the dietary behavior of goitered gazelles, revealing adaptive flexibility that potentially promotes survival under drought conditions [11]. During the spring, goitered gazelle exhibited a relatively broad diet, consuming a diverse array of plant species. This behavior may be attributed to the scarcity of plant resources and the prevalence of short-lived plants in arid and semi-arid regions. According to optimal feeding theory, when food resources are scarce, animals will have a generalized diet (i.e., they will feed on a wider variety of food) [66,67,68]. Goitered gazelle showed elevated values of both the spring food diversity index *H* and niche width *B*. These findings align with the Niche Expansion Hypothesis, suggesting that generalized herbivores may expand their dietary range to compensate for a diminishing availability or quality of preferred food resources [50,69,70]. Additionally, goitered gazelles consumed extensive forbs and Fabaceae, known for their high nutritional value and palatability [71,72,73] in the spring to meet their nutritional needs. This is a critical period for molting, birthing, and nurturing offspring [74], during which goitered gazelles require high-quality food to support essential physiological processes. In the summer, short-lived plants disappear, and graminoids grow rapidly. Fresh stems and leaves have a high water content and are easily digestible. Consequently, the number of plant species consumed by goitered gazelles decreased, with an increased utilization of graminoids. Similarly, a dietary analysis of khulan (*Equus hemionus*) in northern Xinjiang of China, which has a habitat similar to that of goitered gazelles in Luoshan, indicated that they prefer high-nutrient foods in the spring and forge more on graminoids in the summer [75]. In the winter, goitered gazelles also consumed a significant amount of graminoids, as they have high digestibility and can provide sufficient energy to cope with cold conditions. Furthermore, the proportion of trees in the diet was highest in the autumn, while shrubs dominated during the winter. This is likely because there is more leaf litter in the autumn, including leaf litter from *P. sibirica*, *Ulmus glaucescens*, and *S. matsudana*, which are easily found by goitered gazelle. In the winter, due to snow cover, only shrubs and tall grass are easily accessible, explaining the limited variety of plants consumed during this season. When plant resources are scarce, goitered gazelles foraged more on less palatable shrubs to ensure individual survival. The low value of niche width *B* in the winter may be attributed to the need for maintaining a daily energy balance while minimizing the energy expenditure associated with foraging.

Herbivores exhibit specialization in resource-rich environments and generalization in resource-poor conditions [68,76]. Thus, when food is abundant in the summer, goitered gazelles concentrated on foods with higher nutritional value, such as *P. sibirica* and *K. arborescens* [77,78]. This behavior is similar to that of goitered gazelles inhabiting the arid and semi-arid habitats of the Kalamaili Mountain in Xinjiang [28]. In the autumn, characterized by extremely low rainfall in arid regions, water availability influences the survival of goitered gazelles. Consequently, these gazelles primarily sought plants with high moisture contents and good palatability near water sources, such as *S. matsudana* and *N. splendens*. This feeding pattern aligns with the behavior of goitered gazelles and khulan in the Kalamaili Mountain, Xinjiang [27,75]. Abundant crude protein in food can sustain higher enzymatic breakdown rates, enhance fiber digestion, and consequently improve energy utilization [79]. Fabaceae fruits produced in autumn have a high crude protein content [80] and are consumed at high rates by goitered gazelles. However, during autumn, the palatability of Asteraceae plants decreases due to gradual drying and aging, resulting in lower rates of consumption by goitered gazelles than those in spring and summer.

## 5. Conclusions

In the current study, Chenopodiaceae, Rosaceae, Fabaceae, Compositae, and Gramineae formed the primary dietary components, and *Prunus sibirica* and *Krascheninnikovia arborescens* were identified as the key forage plants. The results of this research support the hypothesis that goitered gazelle primarily select forbs, and the degree of selection of major food items differs among seasons. Moreover, the species strategically alters its diet based on variation in the quality and availability of plant resources during different seasons. This adaptive behavior enables goitered gazelle to effectively mitigate seasonal resource bottlenecks. Notably, goitered gazelle distributed in the research area exist as a localized small population, which may have unique ecological characteristics. This results in a relatively small sampling area.

Based on the research results, we proposed several management suggestions. It is recommended to implement measures to maintain the ecosystem of forbs, including controlling excessive grazing and preventing grassland degradation. Additionally, plants with high water and crude protein contents, such as Fabaceae and Chenopodiaceae, are crucial for the survival of herbivores in arid environments. Measures should be taken to protect these plants, such as controlling invasive species, formulating vegetation protection policies, and promoting vegetation regeneration. Finally, ensuring the accessibility and quality of water sources is essential. Measures should be implemented to safeguard water sources from the impacts of over-extraction, pollution, and ecosystem degradation, such as delineating water source protection areas, controlling pollution emissions from agricultural and industrial activities, and restoring and preserving ecosystems. These recommendations are also applicable to other herbivores living in arid and semi-arid regions.

## Figures and Tables

**Table 1:** The food diversity evenness indices and niche width of goitered gazelle were assessed across the four seasons.

	Spring	Summer	Autumn	Winter
Identified species	49	39	42	28
Food diversity index *H*	1.54	1.36	1.34	1.31
Food niche width *B*	21.67	13.94	9.82	6.76

**Table 2:** The relative abundance of principal plant species foraged by goitered gazelle across four seasons.

Plant Functional Groups	Major Plant Species	Relative Density (%)			
		Spring	Summer	Autumn	Winter
Forbs	*Krascheninnikovia arborescens*	14.59	15.50	4.91	18.56
	*Kali collinum*	9.26	5.99	0.42	11.51
	*Aster altaicus*	5.73	3.79	2.09	2.18
Shrubs	*Caragana opulens*	3.65	-	3.34	-
	*Zygophyllum xanthoxylum*	1.46	7.23	2.93	-
Trees	*Prunus sibirica*	2.01	16.53	28.87	17.74
	*Salix matsudana*	1.26	2.48	5.64	-
Graminoids	*Neotrinia splendens*	0.75	1.03	4.39	3.00

## Data Availability

The data presented in this study are available on request from the corresponding author.

## Supplementary Materials

The following supporting information can be downloaded at: https://www.mdpi.com/article/10.3390/ani14050663/s1, Table S1: Food composition of the goitered gazelle during four seasons.
