# Supplementary material for: Seasonal Diet Composition of Goitered Gazelle (Gazella subgutturosa) in an Arid and Semi-Arid Region of Western China
Source: Animals (Basel). 2024 Feb 20;14(5):663. doi: 10.3390/ani14050663 (PMC10931280; doi:10.3390/ani14050663)
Supplement: Supplementary file 1 [file animals-14-00663-s001.zip › Supplementary File.pdf]

Table S1. Food composition of the goitered gazelle during four seasons.

Plant species and family	*RD* (%)			
	Spring	Summer	Autumn	Winter
**Plumbaginaceae**	2.51	3.85	3.34	0
Limonium	2.51	3.85	3.34	0
Limonium bicolor	2.51	3.85	3.34	0
**Liliaceae**	3.34	3.37	0	0
Asparagus	3.34	3.37	0	0
Asparagus brachyphyllus	3.34	3.37	0	0
**Lamiaceae**	5.36	3.27	4.91	6.60
Thymus	2.21	1.10	2.51	5.13
Thymus mongolicus	2.21	1.10	2.51	5.13
Dracocephalum	2.01	0.96	1.88	1.47
Dracocephalum heterophyllum	2.01	0.96	1.88	1.47
Scutellaria	1.13	1.20	0.52	0
Scutellaria baicalensis	1.13	1.20	0.52	0
**Fabaceae**	19.79	4.16	14.31	7.88
Astragalus	1.46	0.79	1.67	0.41
Astragalus melilotoides	1.46	0.79	1.67	0.41
Lespedeza	2.06	0.55	2.09	5.24
Lespedeza davurica	2.06	0.55	2.09	5.24
Hedysarum	1.96	0	1.67	0.71
Hedysarum brachypterum	1.96	0	1.67	0.71
Caragana	9.08	0.41	5.95	0
Caragana roborovskyi	1.51	0.41	1.46	0
Caragana opulens	3.65	0	3.34	0
Caragana sinica	1.96	0	0	0
Caragana korshinskii	1.96	0	1.15	0
Oxytropis	3.69	2.41	1.67	0.27
Oxytropis squammulosa	1.48	1.65	1.15	0.27
Oxytropis aciphylla	2.21	0.76	0.52	0
Senna	1.53	0	1.25	1.25
Senna tora	1.53	0	1.25	1.25
**Gramineae**	1.63	8.60	12.72	14.81
Stipa	0.88	3.69	4.52	7.58
Stipa grandis	0.88	2.93	3.34	2.45
Stipa bungeana	0	0.76	1.18	5.13
Neotrinia	0.75	1.03	4.39	3.00
Neotrinia splendens	0.75	1.03	4.39	3.00
Pennisetum	0	0.83	0.52	0.82

Plant species and family	*RD* (%)			
	Spring	Summer	Autumn	Winter
Pennisetum flaccidum	0	0.83	0.52	0.82
Agropyron	0	1.38	1.78	1.83
Agropyron cristatum	0	0.55	1.78	1.31
Agropyron mongolicum	0	0.83	0	0.52
Eleusine	0	1.69	1.51	1.58
Eleusine indica	0	1.69	1.51	1.58
**Zygophyllaceae**	4.80	10.67	3.76	1.58
Peganum	3.34	3.44	0.84	1.58
Peganum multisectum	1.81	2.06	0	1.31
Peganum harmala	1.53	1.38	0.84	0.27
Zygophyllum	1.46	7.23	2.93	0
Zygophyllum xanthoxylum	1.46	7.23	2.93	0
**Compositae**	15.69	11.15	6.90	6.46
Aster	5.73	3.79	2.09	2.18
Aster altaicus	5.73	3.79	2.09	2.18
Artemisia	8.55	6.06	4.81	4.28
Artemisia stechmanniana	2.39	1.65	0.94	1.23
Artemisia frigida	2.24	1.72	0.84	1.36
Artemisia scoparia	1.66	1.14	0.73	0.82
Artemisia capillaris	2.26	1.55	2.30	0.87
Sonchus	1.03	0	0	0
Sonchus oleraceus	1.03	0	0	0
Taraxacum	0.38	1.31	0	0
Taraxacum mongolicum	0.38	1.31	0	0
**Chenopodiaceae**	23.85	21.49	5.33	30.07
Kali	9.26	5.99	0.42	11.51
Kali collinum	9.26	5.99	0.42	11.51
Krascheninnikovia	14.59	15.50	4.91	18.56
Krascheninnikovia arborescens	14.59	15.50	4.91	18.56
**Polygonaceae**	1.06	0.69	0.84	0
Rheum	1.06	0.69	0.84	0
Rheum racemiferum	1.06	0.69	0.84	0
**Gentianaceae**	3.72	0	0.63	0
Gentiana	3.72	0	0.63	0
Gentiana macrophylla	1.51	0	0	0
Gentiana dahurica	2.21	0	0.63	0
**Apocynaceae**	0.70	1.03	0.84	0
Vincetoxicum	0.70	1.03	0.84	0

Plant species and family	*RD* (%)			
	Spring	Summer	Autumn	Winter
Vincetoxicum mongolicum	0.70	1.03	0.84	0
**Verbenaceae**	1.76	3.37	1.78	10.04
Caryopteris	1.76	3.37	1.78	10.04
Caryopteris mongholica	1.76	3.37	1.78	10.04
**Rosaceae**	5.71	20.83	32.74	18.77
Potentilla	3.70	4.30	3.86	1.04
Potentilla bifurca	1.61	1.55	1.67	1.04
Potentilla acaulis	1.21	2.75	1.46	0
Potentilla sischanensis	0.88	0	0.73	0
Prunus	2.01	16.53	28.87	17.74
Prunus sibirica	2.01	16.53	28.87	17.74
**Brassicaceae**	1.56	0	0	0
Lepidium	0.70	0	0	0
Lepidium latifolium	0.70	0	0	0
Braya	0.85	0	0	0
Braya humilis	0.85	0	0	0
**Scrophulariaceae**	0.70	0	0.42	0
Rehmannia	0.70	0	0.42	0
Rehmannia glutinosa	0.70	0	0.42	0
**Convolvulaceae**	0.75	0.89	0.42	0
Convolvulus	0.75	0.89	0.42	0
Convolvulus tragacanthoides	0.75	0.89	0.42	0
**Linaceae**	0.40	0	0	0
Linum	0.40	0	0	0
Linum perenne	0.40	0	0	0
**Salicaceae**	1.26	2.48	5.64	0
Salix	1.26	2.48	5.64	0
Salix matsudana	1.26	2.48	5.64	0
**Ulmaceae**	1.13	1.03	2.19	2.18
Ulmus	1.13	1.03	2.19	2.18
Ulmus glaucescens	1.13	1.03	2.19	2.18
**Iridaceae**	1.21	1.38	0	0
Iris	1.21	1.38	0	0
Iris lactea	1.21	1.38	0	0
**Rutaceae**	2.26	0	0.73	0
Haplophyllum	2.26	0	0.73	0
Haplophyllum tragacanthoides	1.36	0	0.73	0
Haplophyllum dauricum	0.91	0	0	0

Plant species and family	*RD* (%)			
	Spring	Summer	Autumn	Winter
**Ephedraceae**	0.80	1.72	2.51	1.61
Ephedra	0.80	1.72	2.51	1.61
Ephedra przewalskii	0.80	1.72	2.51	1.61
